# Development of thermosensitive poloxamer 407-based microbubble gel with ultrasound mediation for inner ear drug delivery

**DOI:** 10.1080/10717544.2021.1938758

**Published:** 2021-06-18

**Authors:** Ai-Ho Liao, Cheng-Ping Shih, Ming-Wei Li, Yi-Chun Lin, Ho-Chiao Chuang, Chih-Hung Wang

**Affiliations:** a Graduate Institute of Biomedical Engineering, National Taiwan University of Science and Technology, Taipei, Taiwan; b Department of Biomedical Engineering, National Defense Medical Center, Taipei, Taiwan; c Department of Otolaryngology–Head and Neck Surgery, Tri-Service General Hospital, National Defense Medical Center, Taipei, Taiwan; d Department of Mechanical Engineering, National Taipei University of Technology, Taipei, Taiwan; e Graduate Institute of Medical Sciences, National Defense Medical Center, Taipei, Taiwan; f Taichung Armed Forces General Hospital, Taichung, Taiwan

**Keywords:** Ultrasound, microbubbles, cavitation, thermosensitive gel, poloxamer 407, dexamethasone

## Abstract

Our previous study first investigated feasibility of applying ultrasound (US) and microbubbles (MBs) via external auditory canal to facilitate drug delivery into inner ear. However, most drugs are in aqueous formulae and eliminated via Eustachian tubes after drug application. In this study, feasibility of sustained release of thermosensitive poloxamer 407 (P407)-based MB gel for US mediation-enhanced inner ear drug (dexamethasone, DEX) delivery was investigated. The sol-to-gel transition temperature showed that mixture of DEX and only 10% and 12.5% P407 in MBs can be used for *in vitro* and *in vivo* drug delivery experiments. In *in vitro* Franz diffusion experiments, the release rates of 12.5% P407-MBs + US groups in the model using DEX as the delivered reagent at 3 h resulted in values 1.52 times greater than those of 12.5% P407-MBs groups. In guinea pigs, by filling tympanic bulla with DEX in 12.5% P407-MBs (DEX-P407-MBs), USMB applied at post-treatment days 1 and 7 induced 109.13% and 66.67% increases in DEX delivery efficiencies, respectively, compared to the group without US. On the 28th day after US-mediated P407-MB treatment, the safety assessment showed no significant changes in the hearing thresholds and no damage to the integrity of cochlea or middle ear. These are the first results to demonstrate feasibility of US-modified liquid form DEX-P407-MB cavitation for enhancing permeability of round window membrane. Then, a gel form of DEX-P407-MBs was generated and thus prolonged the release of DEX in middle ear to maintain the therapeutic DEX level in inner ear for at least 7 days.

## Introduction

Intratympanic (IT) delivery of therapeutic agents is often utilized to treat inner ear diseases. Compared with the systemic route for drug delivery, this delivery route has superior characteristics, including the lack of interference from the blood–labyrinth barrier and the lack of systemic adverse effects of drugs (Nyberg et al., 2019). Currently, IT injection is the most commonly used method in clinical practice (McCall et al., 2010). However, repeated injections are usually necessary to achieve the therapeutic effect of drugs. Several materials and devices have been developed to enhance the IT delivery of drugs. Hydrogels are designed to facilitate delivery by increasing the duration of drug contact with the round window membrane (RWM) (Mäder et al., 2018). Hydrogels of poloxamer 407 (P407), a biopolymer of the triblock poloxamer class, have been particularly explored for their potential as drug carriers for the local delivery of dexamethasone (DEX) to the inner ear (Piu et al., 2011). After IT administration, the liquid poloxamer suspension undergoes rapid conversion to a hydrogel matrix capable of providing sustained release of DEX in the inner ear over a period of at least 10 days (Wang et al., 2009). However, based on diffusion characteristics intrinsic to gel-based drug carriers, larger perforations are necessary to enhance the rate of drug delivery into the cochlea (Santimetaneedol et al., 2019). In the present study, instead of perforations on the RWM, ultrasound (US)-mediated P407-based microbubble (MB) cavitation (USMB) for enhancement of inner ear drug delivery was investigated.

Gel-forming hydrogels are a special class of polymeric networks that have several advantages and have been used to overcome the shortcomings of conventional drug formulations in the drug delivery field (Hoffman, 2002). The advantages of hydrogels include the absence of crosslinking agents and the lack of photoirradiation, organic solvents, and heat released during polymerization (Gong et al., 2013). In advanced drug delivery systems, smart hydrogels can reversibly swell and deswell, and the swelling properties are related to the external environment, such as pH, temperature, and ionic concentration, which can, in turn, contribute to collapse or phase change (Ahmed, 2015). Thermosensitive hydrogels are very important biomaterials that can be injected to potentially act as sustained drug release depots *in vivo* with low sol-gel transition temperatures around physiological temperatures (Jeong et al., 2002; Klouda & Mikos, 2008). The sol-gel phase transition property of thermosensitive hydrogels is based on changes in the interaction between hydrophilic and hydrophobic segments in the polymer with water molecules, thus inducing a change in the solubility of the crosslinked network (Huang et al., 2019). Temperature-sensitive hydrogels can be divided into negatively thermosensitive (e.g. N-isopropylacrylamide, cellulose, chitosan, pluronic or poloxamers, and polyethylene glycol) and positively thermosensitive (e.g. gelatin, agarose, amylose, amylopectin, etc.) hydrogels according to their temperature-sensitive structure (Jeong et al., 2002; Huang et al., 2019). Temperature-sensitive hydrogels, which gel at physiological temperature, have been extensively studied due to their potential biomedical applications, including controlled drug delivery, cell encapsulation, and tissue engineering (Lee et al., 2001; Jeong et al., 2002; Gong et al., 2013). However, the disadvantages of temperature-sensitive hydrogels, such as slow temperature response, low mechanical properties, and poor biocompatibility, limit their widespread application (Huang et al., 2019).

The P407 copolymer shows thermoreversible properties, suggesting it may be used to optimize drug formulations (the fluid state at room temperature facilitating administration and gel state above sol-gel transition temperature at body temperature promoting the prolonged release of pharmacological agents) for various applications considering its local inertia and prolongation of drug residence (Dumortier et al., 2006; Soliman et al., 2019). Poloxamer gel has been reported to be of interest in liquid suppository systems, intramuscular drug delivery systems, inner ear drug delivery systems, ocular drug delivery systems, etc. This gel has the potential to be in the liquid state at refrigerated temperatures of 4–5 °C and convert into a gel at body temperature, thereby retarding the release of the drug from the gel (Mendonsa et al., 2018).

The effect of P407 on the middle ear and inner ear has been investigated. P407 can be biodegraded *in vivo* or discharged via Eustachian tubes and causes no inflammation in the middle ear cavity (Feng et al., 2007). Treatment drugs formulated in P407 shared significantly more prolonged exposure than those formulated in aqueous solutions both *in vitro* and *in vivo* in the inner ear (Wang et al., 2011). P407 was also used to prevent chlorhexidine ototoxicity with poloxamer in rats (Dirain et al., 2018). In humans, a P407-based antibiotic formulation (OTIPRIO^®^; Otonomy, Inc.) administered via IT injection has been approved for the treatment of middle ear infections (Mair et al., 2016; Santimetaneedol et al., 2019). Animal studies have demonstrated that P407-based DEX formulations delivered via IT injection allow sustained-release drug delivery to the cochlea (Wang et al., 2009; Salt et al., 2011; Wang et al., 2011). Some studies have confirmed that entry of drugs into the ear can be markedly enhanced with the use of chemical permeation-enhancing agents (Li et al., 2018). Micro perforations in the RWM have been suggested to enhance the rate of drug delivery into the cochlea (Santimetaneedol et al., 2019). However, diffusion of drugs in P407 across an artificial membrane increases with a large perforation but not with multiple small perforations, perhaps due to the diffusion characteristics intrinsic to gel-based drug carriers (Dirain et al., 2018).

Our previous study first demonstrated the feasibility and supported the potential clinical application of US-mediated MB cavitation via the external auditory canal to facilitate drug delivery into the inner ear (Liao et al., 2020). We also assessed the usefulness of US plus US contrast agent MBs in agarose gels for enhancing transdermal drug delivery (Liao, Lu, Hung, et al., 2016). The study revealed that the oscillation behavior and survival of MBs with US are affected by the viscosity of the surrounding medium and that in mice, treatment with US plus MBs in an optimal agarose gel condition can increase skin permeability and enhance transdermal drug delivery. In the present study, potential US-mediated P407-based MB cavitation for inner ear drug delivery enhancement without perforations or chemical permeation-enhancing agents was investigated. The optimal P407-based DEX MBs were first created for noninvasive US-mediated transcanal delivery for inner ear drug delivery enhancements.

## Materials and methods

### Preparation of albumin-shelled MB in dexamethasone-P407 gel

According to the procedure used in our previous studies, albumin-shelled MBs were prepared (Liao, Lu, Lin, et al., 2016). Briefly, albumin-shelled MBs were generated by sonication in 10 mL of a solution containing 132 mg of albumin (Octapharma, Vienna, Austria) and Perfluoropropane (C_3_F_8_) gas (C.C. Gaseous Corporation, New Taipei City, Taiwan) in physiological saline (pH 7.4, 0.9% sodium chloride) using a sonicator (Branson Ultrasonics, Danbury, CT, USA) for 2 min. The number of C_3_F_8_-filled albumin MB in the solution was measured with the MultiSizer III device (Beckman Coulter, Fullerton, CA, USA) using a 30-µm-aperture probe with measurement boundaries of 0.6 and 20 µm. The size distribution in the suspension was measured based on dynamic light scattering (Zetasizer Nano ZS90, Malvern, Worcestershire, UK). There were the albumin-shelled MBs with a mean diameter of 2.7 ± 0.17 μm and a mean concentration of 4.2 ± 0.22 (×10^9^ particles/mL). For the preparation of a DEX (2%)-P407 (10%)-based microbubble gel, P407 (15.63 g; First Chemical, Taipei, Taiwan) was dissolved in 84.37 mL of physiological saline (pH 7.4, 0.9% sodium chloride) and stirred for 30 min at 4 °C and 600 rpm. Since DEX sodium topical 2% gel can be used to treat otic disorders in guinea pigs (Piu et al., 2011), 200 mg of DEX (average molecular weight of 392.464 g/mol; Cyaman Chemical, Ann Arbor, MI, USA) was dissolved in 1 mL of dimethyl sulfoxide (DMSO, 99.7% purity, Sigma-Aldrich, St. Louis, MO, USA). DEX solution (0.1 mL) was added to 0.8 mL of 15.63% P407 solution, and the DEX and P407 solution were adjusted to pH 7 using 2,2′,2′′-nitrilotriethanol (First Chemical). Before the experiments, 0.1 mL of MBs (4.2 × 10^8^ particles) was added to 0.9 mL of the DEX-P407 gel to produce 10% MBs (henceforth referred to as ‘MB’) in 2% DEX and 12.5% P407 gel (henceforth referred to as ‘DEX-P407 gel’).

### Optimization of the composition of the thermosensitive P407 gel for USMB-enhanced middle ear drug delivery by high-frequency US imaging

**Figure 1.**
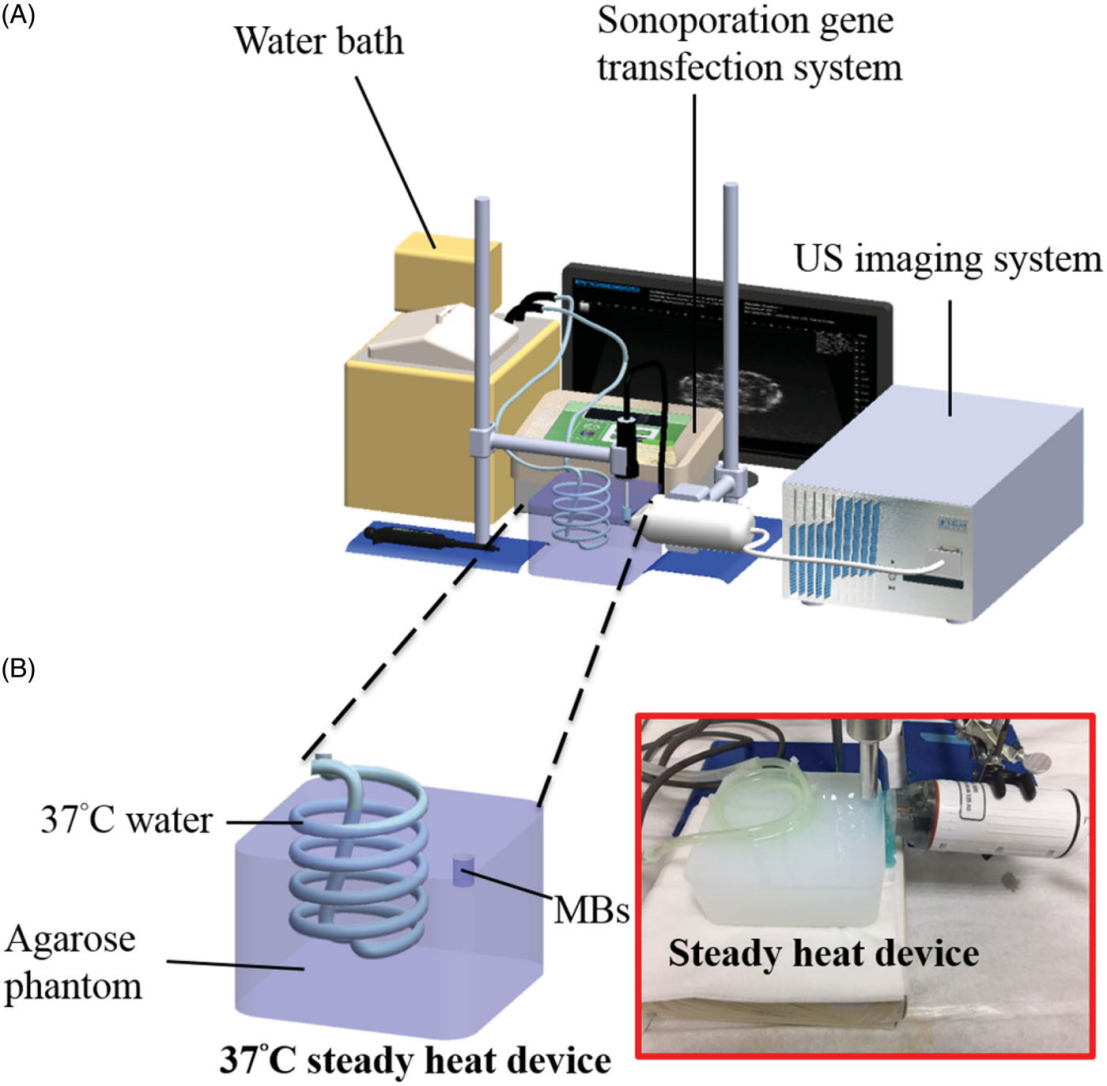
(A) Schematic of the steady tissue-mimicking 37 °C heat setup for measuring US-mediated MB cavitation with a high-frequency ultrasound imaging system. (B) Schematic illustration of a steady tissue-mimicking 37 °C heat device in this study.

For the development of a sustained-release DEX-P407 gel formulation of DEX in inner ear cochlear fluids, high-frequency US imaging (Prospect, S-Sharp Corporation, New Taipei City, Taiwan) was performed to evaluate the effect of different viscosities using P407 for US-mediated MB cavitation, and the experimental setups are shown in Figure 1. A steady tissue-mimicking 37 °C 2% agarose square-column phantom (10 × 20 × 20 mm^3^) was constructed with a 2 × 2 × 20 mm^3^ chamber at its center to load 400 μL of albumin-shelled MB (4.2 × 10^8^ MBs/mL) in P407 gel (2%, 5%, 8%, 10%, 12.5%, 14.5%, 15.5%, 17%). A US transducer with a diameter of 10 mm (ST2000V, Nepagene, Ichikawa, Japan) operated at 1 MHz. The power density was set at 3 W/cm^2^ with a duty cycle of 50% and a pulse repetition period of 250 ms for 1 min. High-frequency US images were received using a transducer with a central frequency of 40 MHz, with axial and lateral resolutions of 30 and 60 µm, respectively. The axial and lateral fields of view were 20 and 20 mm, respectively. Real-time B-mode image planes were obtained with optimization of the gain and the time-gain compensation settings, which were kept constant throughout the experiments. Since the drug delivery enhancement is related to the destruction efficacy of MBs (Liao, Lu, Hung et al., 2016; Liao, Lu, Lin, et al., 2016; Liao et al. 2020). Images were processed with custom MATLAB programs (The MathWorks, Natick, MA, USA) to assess the destruction efficiency of MBs. The region of interest was drawn to calculate the average pre- and post-sonication image intensities in B-mode images.

### The effect of the P407 concentration with or without US on gel formation and rheological evaluation

For simulation of the temperature environment in which samples (with and without US treatments) enter the middle ear cavity, the temperature changes of various concentrations of P407 samples were measured from room temperature to a steady tissue-mimicking 37 °C heat device (the schematic diagram and actual image are shown in Figure 1(B)). To develop suitable formulations using P407 for US-mediated MB cavitation, we first evaluated the impact of the P407 concentration on gel formation. Purified P407 at concentrations of 2%, 5%, 10%, 12.5%, 14.5%, 15.5%, and 17% (w/v) was dissolved in physiological saline and stirred for 30 min at 4 °C and 600 rpm. Then, a temperature sweep and shear rate sweep were performed for each prepared sample with or without US using a rheometer to measure the sol-to-gel transition temperature (*T*_sol-gel_), maximum recorded storage modulus, and viscosity of the P407 samples. Free visible air bubbles in the P407 samples were prepared, and a hood covered the cone-plate geometry to mitigate evaporation. The *T*_sol-gel_ maximum recorded the storage modulus, and viscosity measurements for purified P407 solutions were conducted using a cone-plate geometry (radius 12.5 mm, 0° angle) and a 1000 μL sample volume with an MCR-102 torsional rheometer (Anton Paar) that detects torque (T) in the range 5 to 200 mN. A temperature sweep (10–45 °C) was conducted to record the storage (G’) and loss modulus (G”) of the P407 solutions during gelation. *T*_sol-gel_ was defined as the temperature at which the storage modulus (G’) was halfway between the values of the storage modulus for the solution and the gel (Fakhari et al., 2017). The maximum storage modulus was recorded for each sample as an indication of the strength of the formed gel. The viscosity of the samples was measured over a shear rate sweep of 1–50 s^−1^. The testing was conducted at 10 °C, and the viscosity at a shear rate of 50 s^−1^ was reported to compare the samples.

### In vitro penetration experiments for RWM permeation

*In vitro* RWM penetration was tested and divided into MBs in biotin-fluorescein isothiocyanate (biotin-FITC)-P407 gel and MBs in DEX-P407 gel using static Franz diffusion cells over an area of 2.14 cm^2^ according to the experimental design used in our previous study (Liao et al., 2020). The probe of the sonoporation gene transfection system (1 MHz, 3 W/cm^2^, 50% duty cycle, 1 min, *I*_SPTA_ = 0.655 W/cm^2^; ST 2000 V, NepaGene, Ichikawa, Japan) was positioned 5 mm above an intervening cellulose membrane (1000 kDa; Orange Scientific, Braine-l’Alleud, Belgium). The temperature of the diffusion assembly was maintained at 37 °C. The probe of the sonoporation system and MB in the biotin-FITC-P407 gel or DEX-P407 gel were applied to the donor compartment, which was completely filled with receiving medium consisting of a 1000-µL mixture of biotin-FITC (40 µg/mL; Anaspec, Fremont, CA, USA) or DEX (20 mg/mL), P407 (8%, 10%, 12.5% 14.5% or 15.5%) and MBs (4.20 × 10^7^ MBs/mL) and occluded with Parafilm (Pechiney Laboratory Safety Products and Apparel, Inc., Chicago, IL, USA). The receptor diffusion half-cell facing the dermis side was filled with physiological saline (pH 7.4, 12 mL); it contained a stirring bar at 600 rpm and 0.01% gentamicin to prevent bacterial degradation of the DEX during the penetration process. Instead of MB, the solutions in the diffusion cell were filtered through a 0.2-μm micropore filter (Nalgene, Rochester, NY, USA) or a 0.22-μm micropore filter (Millex, Darmstadt, Germany). Aliquots (200 μL) of receptor solution were taken after various time intervals (0, 4, 30, 60, 120, 180, 240, 300, and 360 min), with the cell then refilled with the same volume of fresh receptor solution. Samples were kept in a freezer until analysis by a fluorometer (Fluoroskan Ascent FL, ThermoLab Systems, Helsinki, Finland).

### Enzyme-linked immunosorbent assays of DEX levels

A commercial competitive enzyme-linked immunosorbent assay (ELISA) kit was used for DEX determination (Neogen Corp., Lansing, MI). The samples from the receptor solution and cochlear perilymph of animals were mixed with enzyme immunoassay (EIA) buffer, and 20 µL was added to a DEX ELISA kit-well microplate (AssayMax^™^, St. Charles, MO) and incubated for 45 min at 37 °C. The plates were washed three times with washing buffer (300 μL) to remove the excess liquid. The chromogen substrate solution (150 μL, Enhanced K-Blue, Neogen, KY, USA) was then added, and the plate was incubated at 37 °C for 30 min or until the optimal blue color density had developed. The appropriate stop solution (50 μL, Red Stop Solution, Neogen, KY, USA) was then added. Colorimetric measurements of DEX were performed at 650 nm using an ELISA reader (Synergy H4 Hybrid Reader; BioTek Instruments, Winooski, VT). A standard DEX calibration curve was constructed to obtain the corresponding concentration of DEX in the samples on the measured absorption peaks. Triplicate measurements were performed for each concentration of DEX. The minimal detectable DEX concentration in the samples was 3 ng/mL.

### Animal study and measurement of DEX levels in the perilymph of the inner ear

Guinea pigs were anesthetized with xylazine (Rompun; Bayer) at 10 mg/kg and ketamine (Imalgene, Merial, Lyon, France) at 80 mg/kg intramuscularly and kept warm with a heating pad. A small incision was performed in the cartilaginous portion of the external auditory canal, and the transcanal approach was executed as described previously (Liao et al., 2020). For the ultrasound microbubble treatment (USM) groups, 200 μL of a mixture of MB, 2% DEX, and 12.5% P407 gel was given through the anteroinferior part of the tympanic membrane into the middle ear cavity by using a syringe with a 25-gauge needle. The US transducer was then placed into the speculum and positioned 5 mm from the tympanic membrane. Saline was injected into the space between the tympanic membrane and the probe, and then a 1-min application of US sonication targeting the middle ear cavity at a power density of 3 W/cm^2^ (acoustic pressure = 0.266 MPa) was performed. These procedures were accomplished under an operating microscope (F-170, Carl Zeiss, Jena, Germany). For the control groups, the middle ear cavity was filled as described above with 200 μL of a mixture of MB, 2% DEX, and 12.5% P407 gel (for the round window soaking (RWS) group) or 2% DEX solution (for DEX only without MBs group).

After the guinea pigs were euthanized with CO_2_ gas, the inner ear perilymph was immediately aspirated. After the tympanic bulla was harvested, a 10-μL microtip on a pipette was inserted through a cochleostomy inferior to the RWM for perilymph aspiration. Subsequently, the collected perilymph was centrifuged immediately, stored at −80 °C, and later processed for the measurement of DEX levels.

### Measurement of the P407 levels in the fluid in the middle ear

The middle ear cavity of the guinea pigs was filled with 200 μL of a mixture of MB, 2% DEX, and 12.5% P407 gel. After treatment, the guinea pigs were euthanized at the indicated time, the temporal bones were quickly removed, and the bullae were opened. The fluid residing on the RWM and the middle ear mucosa was collected for analysis. The measurement of P407 concentration was performed using the cobalt thiocyanate colorimetric method (Mao et al., 2004). Colorimetric measurements of P407 were performed at 624 nm using an ELISA reader.

### Auditory brainstem response recording

The hearing function of the guinea pigs was evaluated by recording auditory brainstem responses (ABRs) as described previously (Liao et al., 2020). After the animals were anesthetized, subdermal needle electrodes were placed at the vertex (positive), below the pinna of the ear (negative), and at the back (ground) of the guinea pigs. Specific stimuli (clicks and 8-, 12-, 16-, 20-, 24-, 28-, and 32-kHz tone bursts) were formed by using SigGen software (Tucker-Davis Technologies, Gainsville, FL, USA) and output monaurally to the external auditory canal via an insert earphone. The average responses from 1024 stimuli at intensities ranging from 5 to 90 dB SPL for each frequency were obtained by decreasing the sound intensity with 5-dB steps. The ABR threshold was defined as the lowest intensity at which a reproducible deflection in the evoked response trace could be identified.

### Distortion product otoacoustic emission measurements

The distortion product otoacoustic emissions (DPOAEs) were measured at center frequencies (FCs) of 8, 16, 20, 24, and 32 kHz with a real-time signal processing system (Tucker-Davis Technologies), as reported previously (Chen et al., 2018). Two simultaneous continuous pure tones, F1 and F2, were calculated using the FC to yield a frequency of two primary tones (Tones 1 and 2). Two separate speakers (EC1 close-field speakers; Tucker-Davis Technologies) were inserted into the animal’s ear canal to generate the two primary tones to elicit DPOAEs. The two primary tones were presented at the same intensity (L1 = L2 = 65 dB SPL) and at a frequency ratio (F2/F1) of 1.2. The DPOAE recordings were measured with a low-noise microphone (ER 10B; Etymotic Research, Elk Grove Village, IL) and averaged 512 times at each frequency. The peak of the cubic difference distortion product (2F1–F2) at different FCs was accepted as a DPOAE if it was 3 dB above the noise floor, and the difference was referred to as the signal-to-noise ratio (SNR).

### Histological examination of the tympanic membrane and middle ear

The guinea pigs were perfused intracardially with 4% buffered paraformaldehyde in PBS and euthanized, and then, their temporal bones were quickly removed. The bullae were opened and fixed in 4% paraformaldehyde for 1 h at room temperature. After fixation, the bullae, including cochleae, were decalcified with 10% ethylenediaminetetracetic acid (EDTA), pH 7.3, at 4 °C on rotation. The tympanic membrane and the surrounding part of the middle ear cavity were separated from the bullae for histological examination, and cochleae were carefully preserved for cochlear surface preparation. The samples were dehydrated through a graded series of ethanol solutions, cleared in xylene, and embedded in paraffin. Paraffin-embedded tissue blocks were cut into 3-mm-thick sections. The sections were stained with hematoxylin and eosin (H&E) (Muto Pure Chemicals Co., Ltd., Tokyo, Japan). The slides were examined using an Olympus BX50 microscope. All images were taken from the slides by a camera (Diagnostic Instruments SPOT-RT SE Digital Camera) connected to the microscope.

### Cochlear surface preparation and immunofluorescence analysis of dexamethasone uptake

The cochleae were perfused and fixed with 4% paraformaldehyde for 1 h at room temperature and then decalcified. After PBS rinse, the organ of Corti was then carefully isolated from the bone surrounding the organ of Corti. The samples were incubated with Alexa Fluor 488-conjugated phalloidin (Thermo Fisher Scientific, Eugene, OR, USA) for 30 min at room temperature, rinsed with PBS, mounted in DAPI Fluoromount-G^®^ mounting medium (Southern Biotech, Birmingham, AL, USA), and covered with a coverslip for the analysis. For DEX immunofluorescence, the fixed cochlear tissues were incubated with PBS containing 0.3% Triton X-100 for 30 min and then blocked with BlockPRO blocking buffer (Visual Protein Biotechnology, Taipei, Taiwan) for 60 min at room temperature. The tissues were then incubated with anti-DEX polyclonal antibodies (1:100; OriGene Technologies, Rockville, MD, USA) and anti-myosin 7a polyclonal antibodies (1:100; Novus Biologicals) overnight at 4 °C. After PBS rinse, the tissues were incubated with Alexa Fluor 488-conjugated donkey anti-sheep antibodies (1:500; Thermo Fisher Scientific) and Alexa Fluor 555-conjugated goat anti-rabbit antibodies (1:500; Thermo Fisher Scientific) for 1 h at room temperature and then incubated with Alexa Fluor 488-conjugated phalloidin for 30 min, mounted in DAPI Fluoromount G^®^ mounting medium. Fluorescence images were acquired using a confocal laser scanning microscope (Zeiss LSM 880, Carl Zeiss, Jena, Germany). Immunostaining was quantified by analyzing all images using ImageJ bundled with 64-bit Java 1.8.0_172 (https://imagej.nih.gov/ij/download). The staining intensities were expressed in arbitrary units (AU) for the different cochlear turns and subjected to histogram analysis.

### Statistical analysis

The obtained data were analyzed statistically using two-tailed Student’s *t*-test for comparisons between two groups. Multiple groups were compared using one-way ANOVA followed by Tukey’s multiple-comparisons test. A probability value of *p* < .05 was considered indicative of a significant difference. Data are expressed as the mean ± standard error of the mean.

### Study approval

This animal study was approved by the Institutional Animal Care and Use Committee of the National Defense Medical Center, Taipei, Taiwan. The pigmented male guinea pigs (weighing 250–350 g) were used for experiments and cared for in accordance with protocols approved institutionally.

## Results

### Characterization of albumin-shelled MB in P407 gel

**Figure 2.**
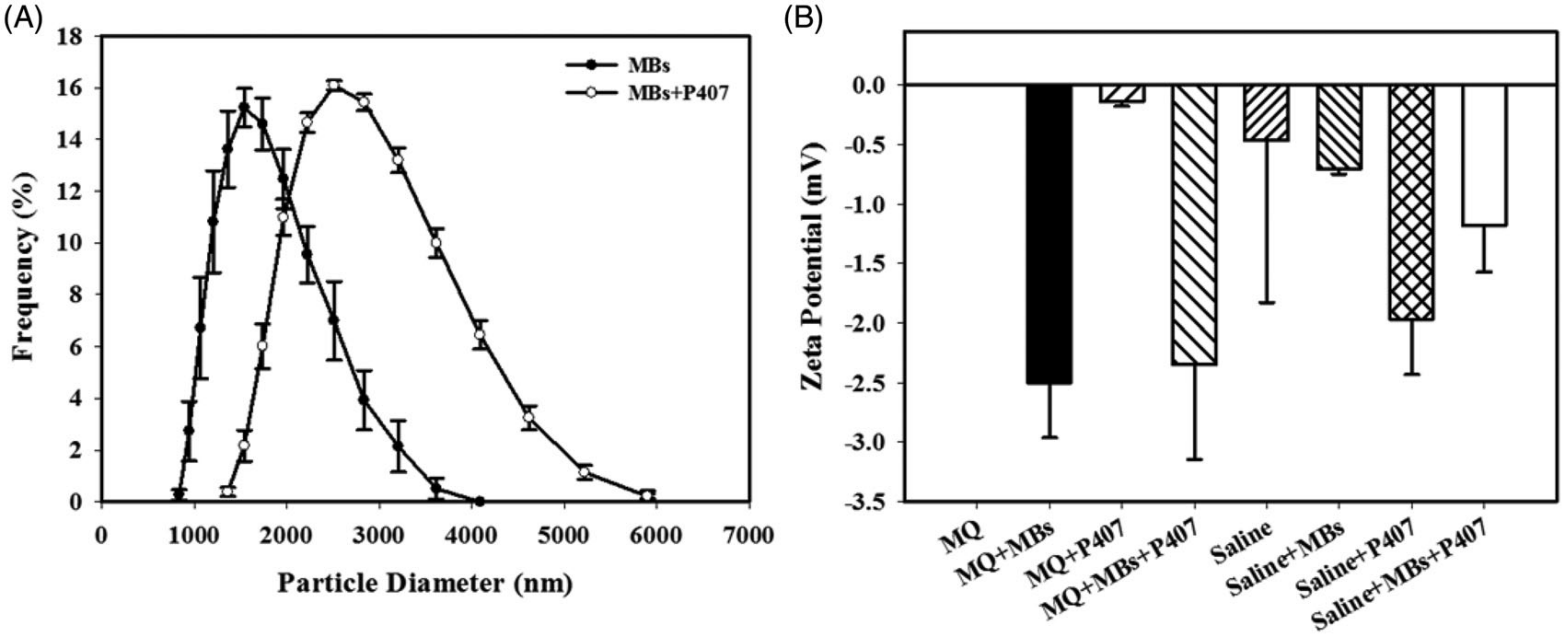
Quantification of the size distributions (A) and zeta potentials (B) of MQ water, MBs in MQ water, P407 in MQ water, MBs and P407 in MQ water, saline, MBs in saline, P407 in saline, and MBs and P407 in saline. Data are the mean and SEM.

The diameters of the albumin-shelled MBs and albumin-shelled MBs in the P407 gel were 1737 ± 113 and 2799 ± 375 nm, respectively (Figure 2(A)); the corresponding concentration of MBs was 4.20 ± 0.223 × 10^9^ bubbles/mL (*n* = 6). In Figure 2(B), the zeta potential of the MBs in Milli-Q (MQ) water (–2.52 ± 0.79 mV) is close to that of the MBs in P407-MQ water (–2.42 ± 0.40 mV). The zeta potential of the MBs in saline at −0.70 ± 0.07 mV was slightly different from that of the MBs in P407 at −1.18 ± 0.40 mV (*n* = 15).

### Optimizing the composition of thermosensitive P407 gel in vivo for middle-ear USMB cavitation

**Figure 3.**
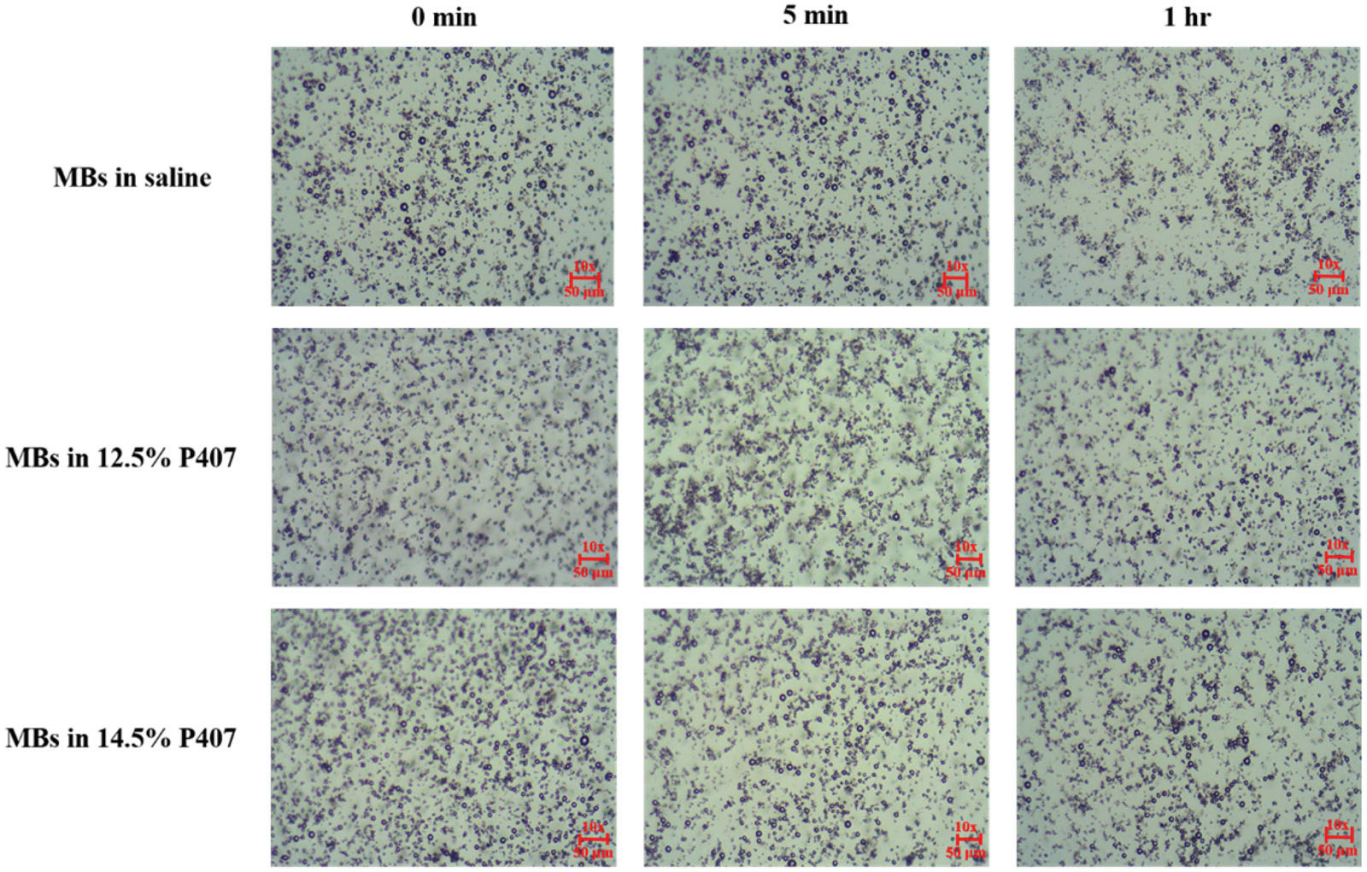
Microscopy images of MBs in saline and 12.5% and 14.5% P407 gels after 5 min and 1 h.

**Figure 4.**
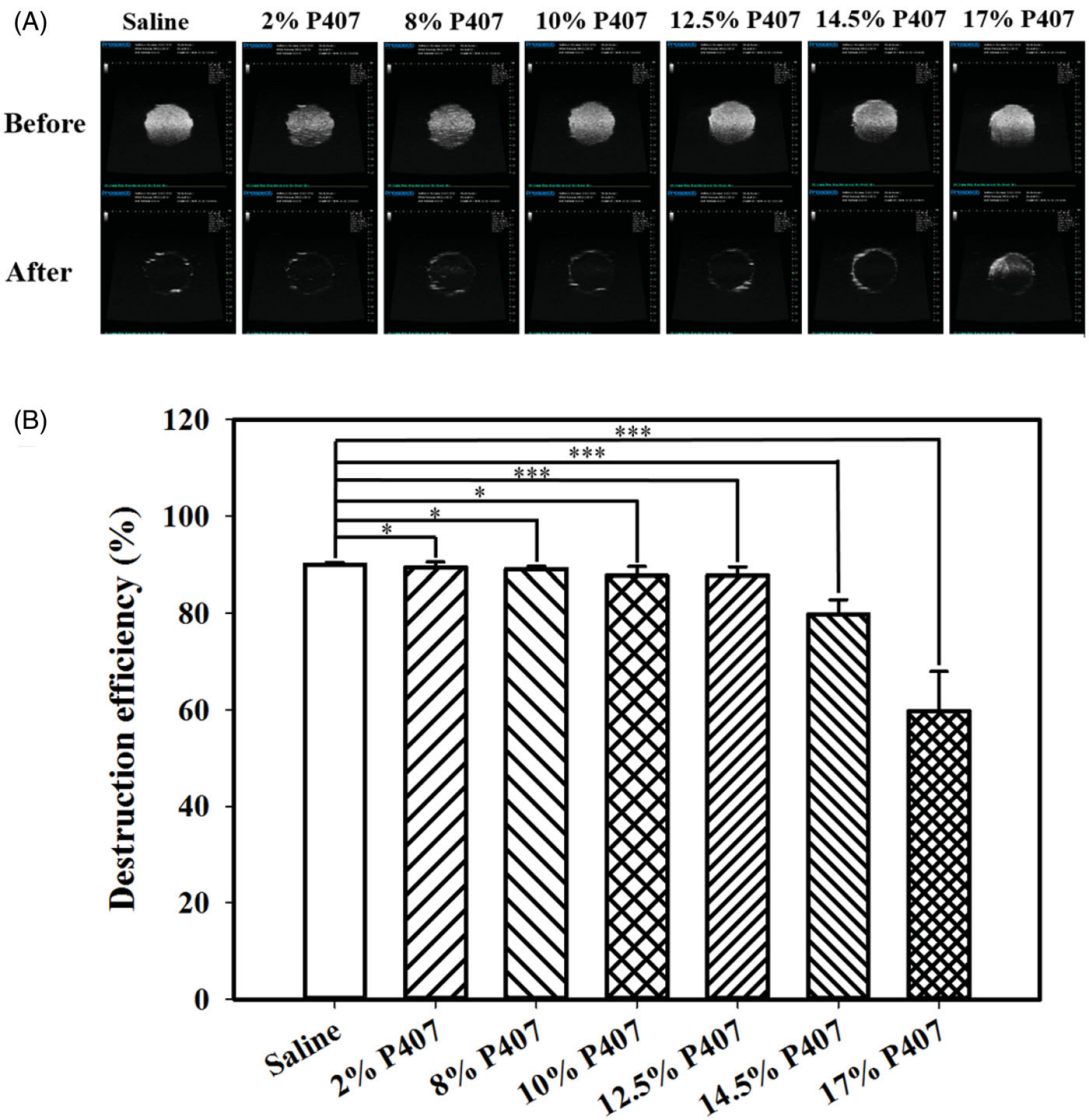
(A) High-frequency US images of MBs before and after US sonication. (B) Quantification of the disruption efficiency. Values are expressed as the mean ± SEM (*n* = 5 per group). US, ultrasound; MBs, microbubbles. *p < .05; **p < .01; ***p < .001; #*p* < .001 relative to the control group; one-way ANOVA followed by Tukey’s multiple-comparisons test.

Figure 3 shows microscopy images of MB in saline and in the 12.5% and 14.5% P407 gels after standing for 5 min and 1 h. The images indicate that placing MB in the 12.5% and 14.5% P407 gels either for 5 min or for 1 h did not destroy them (Liao et al., 2018). High-frequency US images of MBs in saline and 2%, 8%, 10%, 12.5%, 14.5%, and 17% gels without and with US sonication at 3 W/cm^2^ for 1 min are shown in Figure 4(A). In Figure 4(B), the efficiencies of MB destruction for US sonication at power densities of 3 W/cm^2^ for 1 min were 89.97%, 89.42%, 89.09%, 87.70%, 87.77%, 79.75%, and 59.77% (*n* = 5). For 5 min of observations, the temperatures of MB in saline and 2% and 8% P407 in the chamber of the phantom with and without US sonication in a steady tissue-mimicking 37 °C heat device increased more significantly than in other concentrations of P407.

### The effect of P407 concentration on gel formation and MB, DEX, or US addition to thermogelling properties

**Figure 5.**
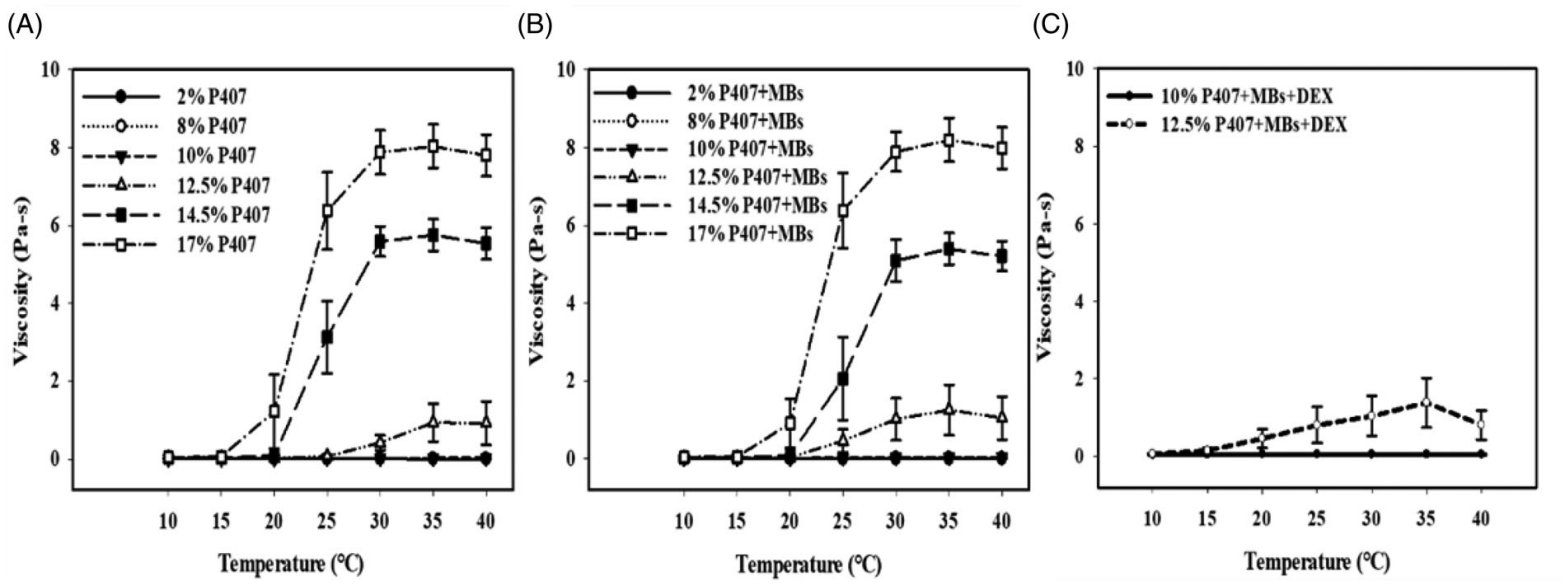
The impact of (A) P407 concentration, (B) MB addition, and (C) DEX addition on gel formation.

The impact of P407 concentration on gel formation at various temperatures is shown in Figure 5(A). Figure 5(A) indicates that no gel was formed at 2% w/v, 8% w/v and 10% w/v P407 concentrations. The *T*_sol-gel_ values of 12.5%, 14.5%, and 17% P407 were measured according to Figure 5(A). In Table 1, P407 at higher concentrations underwent a sol-to-gel transition at lower temperatures. Figure 5(B,C) indicate that the viscosity was not impacted by the addition of MBs or MBs and DEX. However, in Table 1, the addition of MBs or MBs and DEX reduced the *T*_sol-gel_ of P407. The *T*_sol-gel_ of 12.5% P407 in MB solution was close to that of P407 in MB and DEX solutions. The gelation temperatures of 14.5% and 17% P407 were low, and it was difficult to add MBs and DEX for tympanic injection. Only 10% and 12.5% P407 in MBs and DEX solution were used in the *in vitro* permeation drug delivery experiments. In a steady tissue-mimicking 37 °C heat device, the heat transfer effect increased significantly and was proportional to the concentrations of P407 (Supplementary Figure 1). However, after US treatment, the heat transfer effect decreased significantly at higher concentrations (10%, 12.5%, 14.5%, and 17%) of poloxamer 407.

**Table 1.** Recorded *T*_sol-gel_ values of four concentrations of P407, P407 in MB solution, and P407 in MB and DEX solution samples.

P407 (%)	*T*_sol-gel_ (°C)	MB addition *T*_sol-gel_ (°C)	MBs and DEX addition *T*_sol-gel_ (°C)
10.0	N/A	N/A	N/A
12.5	30.7	26.9	27.0
14.5	24.2	24.4	N/A
17.0	24.0	23.8	N/A

### In vitro permeation of drugs under optimal P407 conditions

**Figure 6.**
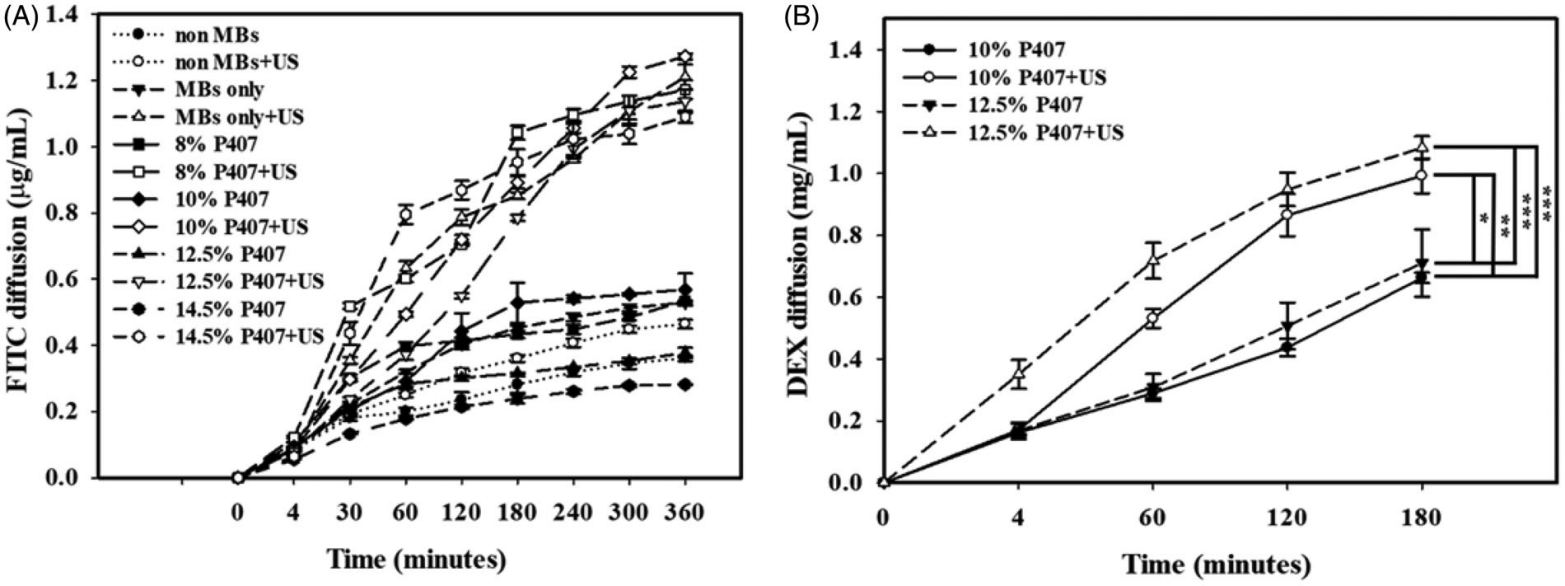
*In vitro* (A) biotin-FITC and (B) DEX penetration in the different experimental groups through a micropore filter in a Franz diffusion cell at 36–37 °C. Data are the mean and SEM; two-tailed Student’s *t*-test and one-way ANOVA followed by Tukey’s multiple-comparisons test.

Figure 6(A) shows the biotin-FITC concentrations in the six groups for synthetic cellulose membrane penetration over 6 h as analyzed using a fluorometer. The concentration in most groups increased rapidly during the first 3 h and then gradually leveled off from 3 to 6 h. After addition of MBs, the concentrations of biotin-FITC were higher in the groups with US than in the groups without US. At 6 h, the concentrations were significantly higher (*p <* .05) in the groups with 10% P407 + US (10% P407 + MBs + US, 1.27 ± 0.09 µg/mL), MBs only + US (1.21 ± 0.04 µg/mL), 8% P407 + US (8% P407 + MBs + US, 1.17 ± 0.03 µg/mL), 12.5% P407 + US (12.5% P407 + MBs + US, 1.14 ± 0.04 µg/mL), and 14.5% P407 + US (14.5% P407 + MBs + US, 1.09 ± 0.02 µg/mL) than in the groups with 10% P407 (10% P407 + MBs, 0.57 ± 0.05 µg/mL), 8% P407 (8% P407 + MBs, 0.53 ± 0.01 µg/mL), MBs only (0.53 ± 0.01 µg/mL), non-MBs + US (0.46 ± 0.01 µg/mL), 12.5% P407 (12.5% P407 + MBs, 0.38 ± 0.02 µg/mL), non-MBs (0.36 ± 0.01 µg/mL) and 14.5% P407 (14.5% P407 + MBs, 0.28 ± 0.01 µg/mL). In Figure 6(B), the concentrations of DEX in the 12.5% P407 + MBs + US group at each time point were significantly higher than those in the other groups (*p <* .001). At 1, 2, and 3 h, the concentrations of DEX in the 10% P407 + MBs + US group were significantly higher than those in the groups with 12.5% P407 + MBs and 10% P407 + MBs (*p <* .002*, p <* .009). The DEX concentrations in the 12.5% P407 + MBs + US and 10% P407 + MBs + US groups were gradually approached with time. At 3 h, the concentrations in the 12.5% P407 + MBs + US, 10% P407 + MBs + US, 12.5% P407 + MBs, and 10% P407 + MBs groups were 1.08 ± 0.08, 0.99 ± 0.13, 0.71 ± 0.24 and 0.66 ± 0.04 mg/mL, respectively. The 12.5% P407 + MBs + US group exhibited the highest permeation of DEX, so 12.5% P407 + MBs was used in the subsequent *in vivo* experiments.

### P407-MB gel prolonged drug residence in the middle ear and facilitated the delivery of drugs into the inner ear under US sonication

**Figure 7.**
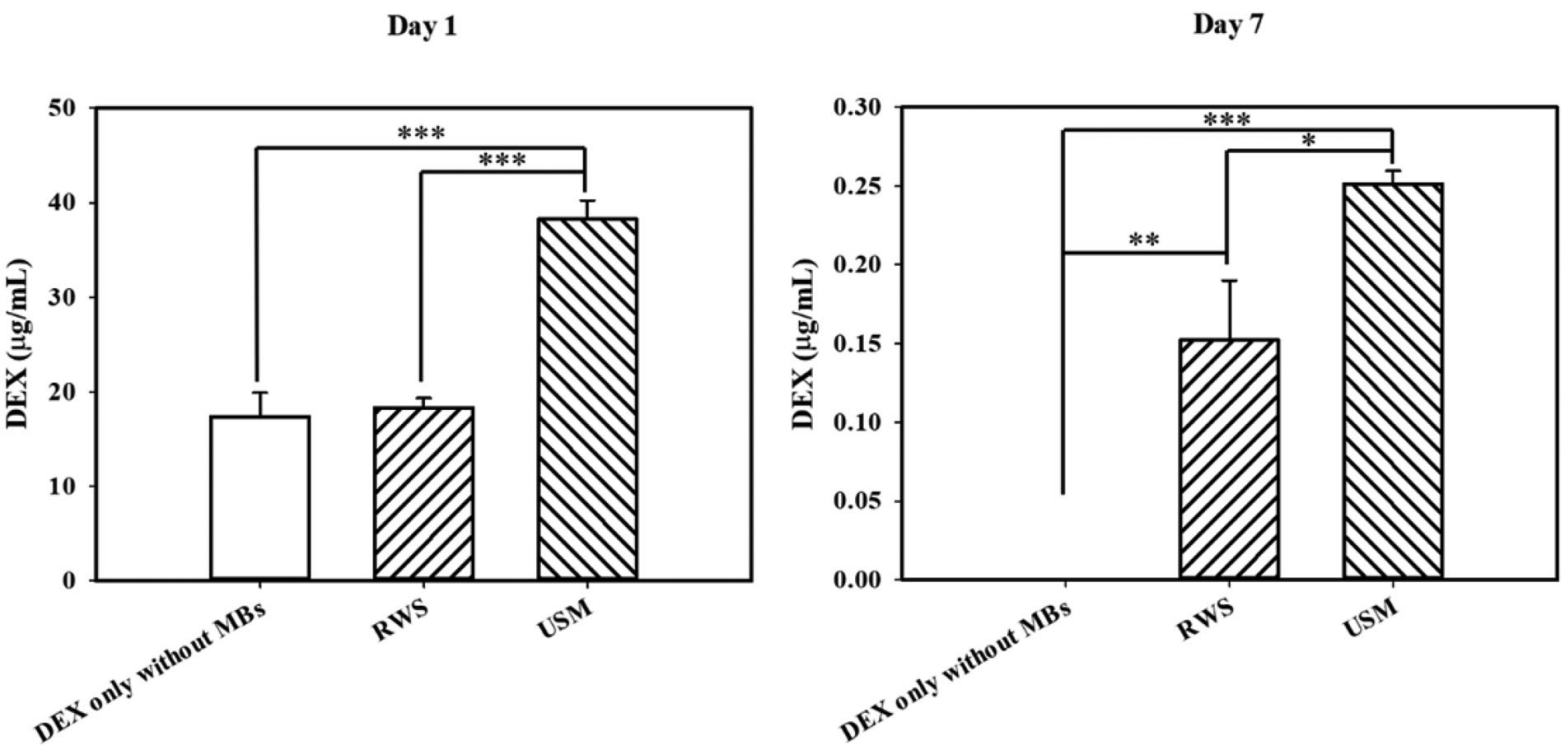
DEX concentrations in the perilymphatic fluid of the inner ear of the groups with USM, RWS and DEX injection without MB at 1 and 7 days after treatment. USMB significantly increased the DEX level of perilymph compared to that of the groups with DEX only with MB and RWS at post-treatment day 1. On post-treatment day 7, the USM group still showed significantly higher DEX levels than the other treatment groups. All graphs represent the mean ± SEM. One-way ANOVA and Tukey’s multiple comparisons test (*n* = 6 each group). *p < .05.

**Figure 8.**
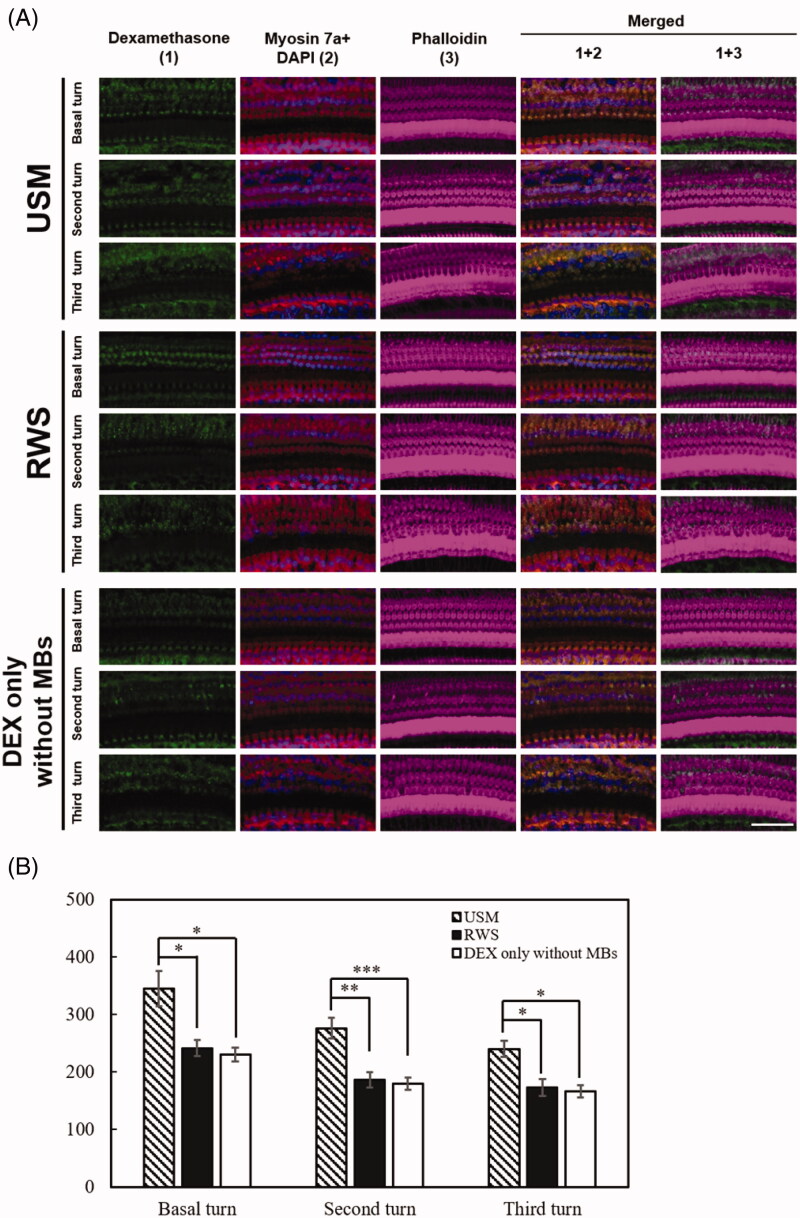
Exposure to the US-mediated P407-MB gel enhances the cellular uptake of DEX in the cochlea. Samples were obtained from the cochleae 1 day after DEX treatments. (A) Representative confocal microscopic images of indirect immunofluorescence staining showing stronger localization of DEX uptake (green) in the USM group than in the groups with RWS and DEX only without MBs in the basal, second, and third turns. Four repetitions of this experiments were conducted. Myosin 7a, cell bodies (red); phalloidin, stereocilia bundles (magenta); DAPI, nuclei (blue). (B) Histogram representations of the mean fluorescence intensity of DEX staining. Data are shown as the means ± SEM (*n* = 4 for each bar). Scale bar: 50 μm. USM, ultrasound microbubble treatment; RWS, round window soaking; MBs, microbubbles; DEX, dexamethasone.

**Figure 9.**
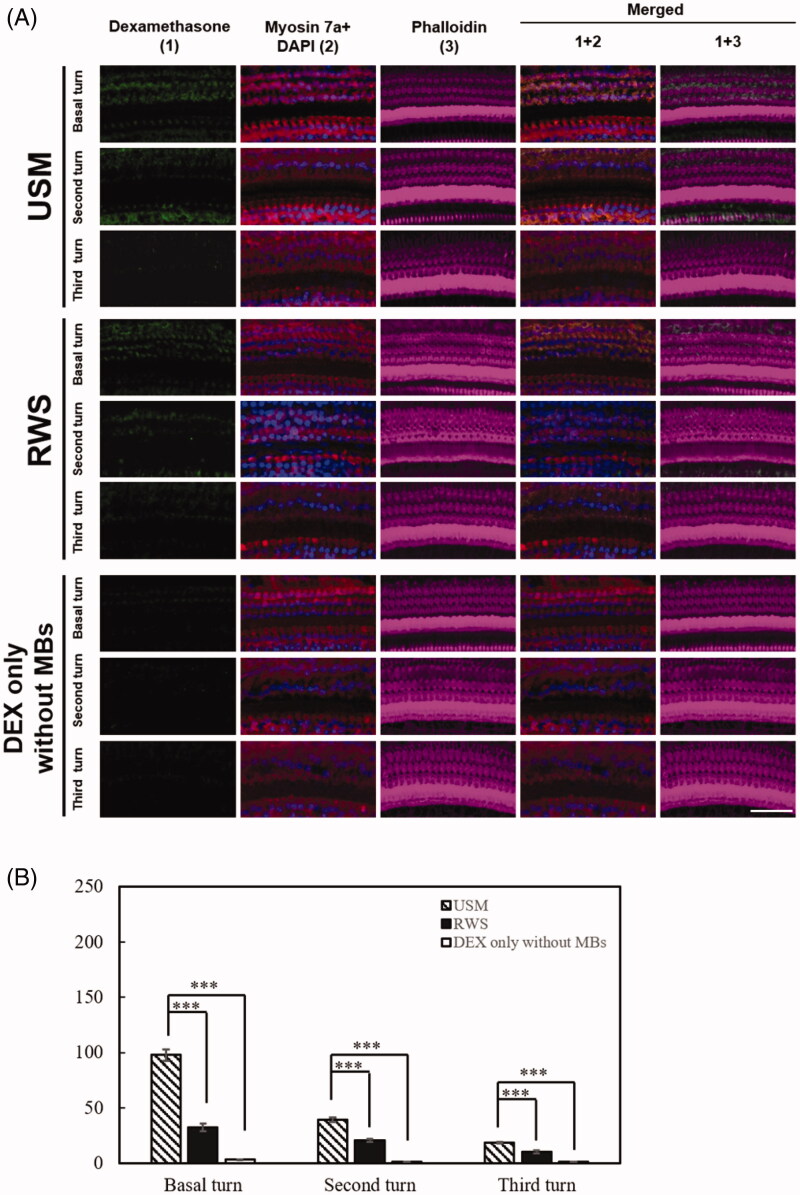
Exposure to the US-mediated P407-MB gel maintains the cellular uptake of DEX in the cochlea for one week. Samples were obtained from the cochleae 7 days after DEX treatments. (A) Representative confocal microscopic images of indirect immunofluorescence staining show stronger localization of DEX uptake (green) in the USM group than in the groups with RWS and DEX only without MBs in the cochlea. Four repetitions of this experiments were conducted. Myosin 7a, cell bodies (red); phalloidin, stereocilia bundles (magenta); DAPI, nuclei (blue). (B) Histogram representations of the mean fluorescence intensity of DEX staining. Data are shown as the means ± SEM (*n* = 4 for each bar). Scale bar: 50 μm. USM, ultrasound microbubble treatment; RWS, round window soaking; MBs, microbubbles; DEX, dexamethasone.

To verify the efficiency of *in vivo* US-mediated P407-MB gel drug delivery to the inner ear system, we used DEX as the delivery drug in the guinea pig model. Based on the *ex vivo* results, DEX-12.5% P407-MB gel was applied for the experiments *in vivo*. The results for the inner ear perilymph are shown in Figure 7. One day after treatments, the DEX concentrations in the groups with USM, RWS, and DEX only without MBs were 38.25 ± 1.96, 18.29 ± 1.05, and 17.38 ± 2.51 μg/mL, respectively. The perilymphatic DEX levels in the USM group were significantly higher than those in the groups with RWS and DEX only without MBs (USM vs. RWS, *p* < .001 and USM vs. DEX only without MBs, *p* < .001). There was no significant difference in the DEX levels between the groups with RWS and DEX only without MBs (*p* = 1). The application of US to the mixture of DEX and P407-MB gel increased the delivery of DEX into the inner ear by approximately 2.1-fold when the RWM was soaked with this gel mixture and 2.2-fold when intratympanic DEX was injected. These findings demonstrated that exposure to US-mediated P407-MB gel in the middle ear cavity facilitated the delivery of drugs into the inner ear. Subsequently, we investigated whether this P407-MB gel could increase the residence time of drugs in the middle ear to attain a sustained therapeutic level of drugs in the inner ear. At 7 days after treatment, the cochlear perilymphatic DEX concentrations in the groups with USM, RWS, and DEX only without MBs were compared. In addition, the fluid in the middle ear among the three groups was collected to assess DEX residence in the middle ear. Seven days after treatment, the perilymphatic DEX concentrations in the USM and RWS groups were 0.25 ± 0.02 and 0.15 ± 0.09 μg/mL, respectively. DEX in the cochlear perilymph of the groups with DEX only without MBs was undetectable (less than 3 ng/mL). At post-treatment day 7, perilymphatic DEX levels in the USM group were significantly higher than those in the groups with RWS and DEX only without MBs (USM vs. RWS, *p* < .041 and USM vs. DEX only without MBs, *p* < .002). On post-treatment day 7, some residual fluid remaining on the RWM was found in the USM and RWS groups, but no fluid was found in the group with DEX only without MBs (Supplementary Figure 2). The DEX concentration of the residual middle ear fluid in both the USM and RWS groups was 23.4 ± 13.03 ng/mL. The P407 concentration of the residual fluid was 10.53 ± 0.13 mg/mL (Supplementary Figure 3). These findings demonstrated that the P407-MB gel can prolong the duration of drug residence in the middle ear. Previous studies have reported that more than 0.001 μg/mL DEX in the inner ear can reduce noise-induced outer hair cell loss, and a level greater than 0.04 μg/mL DEX can achieve a therapeutic effect within the inner ear (Takemura et al., 2004; Piu et al., 2011). On post-treatment day 7, the perilymphatic DEX concentrations in both the USM and RWS groups were over 0.04 μg/mL, and the USM group had a higher DEX level than the RWS group. Therefore, the P407-MB gel extended the exposure of DEX to a therapeutic level in the inner ear for at least one week. Even at 7 days after treatments, the US-mediated P407-MB gel could result in a higher perilymphatic DEX level than the P407-MB gel alone. According to the abovementioned results, the US-mediated P407-MB gel can not only immediately facilitate the delivery of drugs into the inner ear but also provide a sustained release of drugs from the middle ear. Subsequently, one and 7 days after exposure to the US-mediated P407-MB gel, the cochlear tissue was harvested to analyze the uptake of DEX by observation with a confocal laser scanning microscope. On post-treatment day 1, the USM group exhibited strong fluorescence of DEX labeling in the inner and outer hair cells in all turns of the cochlea, especially in the basal turn (Figure 8). Compared to the USM group, the groups with RWS and DEX only without MBs had weaker DEX labeling in all turns. Seven days after treatment, the fluorescence intensity of DEX labeling in the three groups was obviously decreased compared to that at post-treatment day 1 (Figure 9). However, the USM group still exhibited the most intense fluorescence of DEX labeling in the region of hair cells among the three groups. Almost no fluorescence was detected in all turns of the DEX group without MBs. These results demonstrated that US-mediated P407-MB gel enhances and maintains the uptake of DEX by hair cells for at least one week.

**Figure 10.**
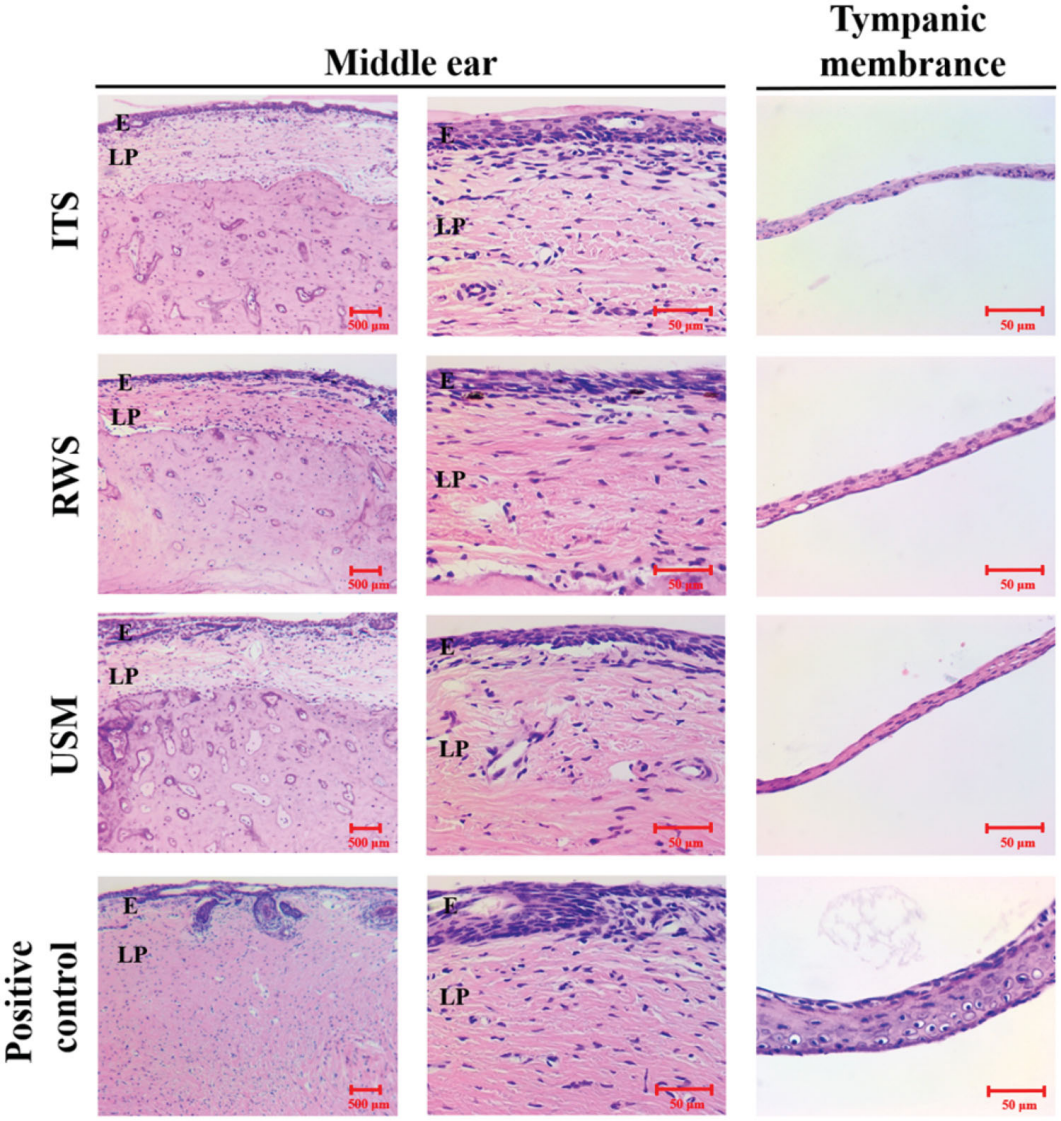
Representative images of immunohistochemical staining (H&E staining) in the middle ear cavity and tympanic membrane of guinea pigs 28 days after USMB treatment. Positive controls treated with one intratympanic injection of 200 μL of LPS (200 μg/mL) presented with acute otitis media with effusion and were sacrificed on post-treatment day 7. ITS, intratympanic saline injection without MBs; E, epithelium; LP, lamina propria.

**Figure 11.**
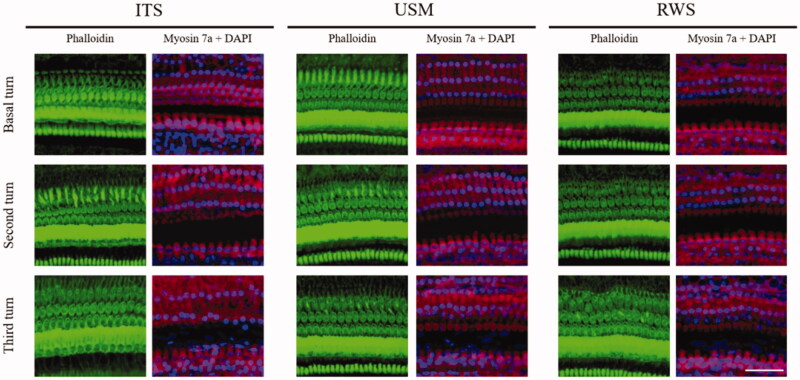
Representative images of confocal microscopic immunofluorescence analysis of cochlear surface preparation 28 days after USMB treatments. There was no difference in the survival of cochlear hair cells between the USM, RWS and ITS groups. The staining shows the nuclei (blue, DAPI), filamentous actin (green, phalloidin), and cell bodies (red, myosin 7a). Four repetitions of this experiments were conducted. Scale bar: 50 μm.

**Figure 12.**
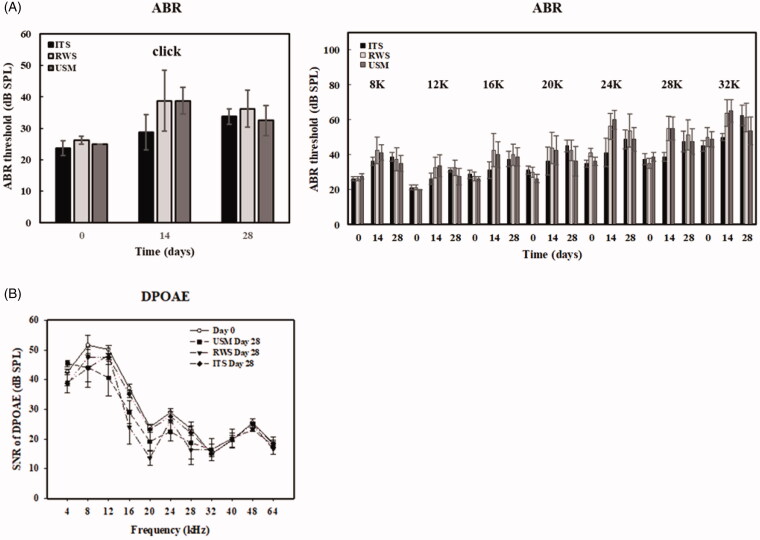
Hearing assessment in guinea pigs after USM treatments. (A) The auditory brainstem response (ABR) threshold recording with click and tone burst stimuli before (day 0) and at a 1-month follow-up in USM, RWS and intratympanic saline injection without MBs (ITS). groups. The results are expressed as the mean ± standard error of the mean (SEM), with *n* = 4 for each bar. (B) Signal-to-noise ratios (SNRs) of the cubic difference distortion product (2F1–F2) at different center frequencies (FCs) for each group. The results are expressed as the mean ± SEM, with *n* = 4 for each group; one-way ANOVA followed by Tukey’s multiple-comparisons test.

Finally, functional and anatomic evaluation of the middle and inner ear related to this technique was investigated to assess its safety. The integrity of the tympanic membrane and ossicles following treatment with the US-mediated P407-MB gel or the P407-MB gel alone was inspected using an operating microscope. No perforation of the tympanic membrane or erosion of the ossicles was found in the USM and RWS groups at 4 weeks after treatment (Supplementary Figure 4). Moreover, in both groups, ossicular mobility was not decreased, and no fibrotic bands were found in the middle ear cavity. Histological examination of the middle ear in the USM and RWS groups was performed and compared to that in the group with intratympanic saline injection without MBs (ITS) and positive controls [a model of acute otitis media with effusion in guinea pigs treated with one intratympanic injection with 200 μL of LPS (200 μg/mL)] (Figure 10). The H&E-stained sections in positive controls showed thickening of the middle ear epithelium and tympanic membrane and many inflammatory cells in the middle ear mucosa. In the ITS, USM, and RWS groups, the thicknesses of the middle ear mucosa and tympanic membrane were similar. No prominent infiltration of inflammatory cells was observed in these three groups. These findings confirmed that exposure of the middle ear cavity to US-mediated P407-MB gel would not lead to any adverse tissue reaction in the middle ear. The loss of cochlear hair cells in the organ of Corti was investigated after exposure to US-mediated P407-MBs (Figure 11). The results indicated that there was no significant hair cell loss in all turns of the cochlea at 4 weeks following exposure. The effect of US-mediated P407-MB gel on the integrity of the RWM was investigated. The RWM consists of the outer epithelial layer, the middle connective tissue layer, and the inner epithelial layer. The confocal microscopic results revealed no significant difference in the cell arrangement of these three layers between the USM, RWS, and ITS groups (Supplementary Figure 5). The outer epithelial layer was intact and had no loss of epithelial cells after exposure to US-mediated P407-MBs in the USM group. It suggested that the integrity of the RWM was preserved after exposure to US-mediated P407-MBs. Functional evaluation of hearing showed no significant difference in the ABR threshold 4 weeks after exposure to US-mediated P407-MBs in the USM group, as well as no differences in ABR thresholds among the USM, RWS, and ITS groups (Figure 12(A)). The SNR of DPOAEs also revealed no significant difference at frequencies from 4 kHz to 64 kHz among these three groups (Figure 12(B)). These data suggested that exposure to US-mediated P407-MBs does not cause either structural or functional damage in the inner ear.

## Discussion

Several drugs and other reagents, including neurotrophins, genetic material, and small molecule drugs, via IT delivery have been shown to have therapeutic effects on the inner ear in animal models. Currently, steroids are one of the main drugs for inner ear therapy (Devare et al., 2018). IT DEX is indicated as a second-line treatment for sudden sensorineural hearing loss refractory to systemic steroids and is considered a first-line treatment in patients with contraindications for oral steroids (El Sabbagh et al., 2017). The injection course of IT DEX is often performed twice or three times a week for 2–4 weeks. IT DEX can be given in patients with Meniere’s disease to control symptoms of vertigo and hearing loss (Espinosa-Sanchez & Lopez-Escamez, 2016). In IT applications, RWM permeability and elimination via the Eustachian tube are two crucial factors for the efficiency of drug delivery into the inner ear (Salt & Plontke, 2018). Since most drug formulations are aqueous solutions, they are easily drained through the Eustachian tube, vasculature, and lymphatic pathways, resulting in drug diffusion to the inner ear for a short time. Several materials and devices have been developed to prolong drug retention in the middle ear, including hydrogels, copolymers, nanocarriers, PLGA bioabsorbable stents, and active IT drug delivery systems (Swan et al., 2008; Hao & Li, 2019). P407 hydrogel is a thermogelling agent and is commonly used for controlled drug release to the inner ear (Mäder et al., 2018). We first demonstrated that USMB targeting of RWM can effectively enhance inner ear drug delivery without damaging the cochlear structure and hearing (Shih et al., 2013). US triggers cavitation of MBs, including inertial and stable cavitation, to produce microstreaming and shock waves in the middle ear to facilitate drug diffusion through RWM. RWM permeability is immediately increased following USMB treatment, and the enhancement of RWM permeability can persist for 72 h after USMB treatment (Shih et al., 2013; Lin et al., 2019). Electron microscopic observation of the RWM revealed heterogeneous pore-like openings and perforation on the outer epithelial layer of the RWM, indicating an acoustic cavitation-induced sonoporation effect on the RWM (Lin et al., 2019). The disruptions in the outer epithelial layer were effectively repaired, and the RWM was completely recovered within 1 month post-USMB (Lin et al., 2019). Moreover, we demonstrated that the efficiency of DEX delivery into the inner ear can be enhanced by USMB. In an animal model of noise-induced hearing loss, the group with combined treatment of DEX and USMB showed better hearing preservation, cochlear protection, and anti-inflammatory effects of DEX than the control groups (Shih et al., 2019). However, the mixture of DEX and MBs is aqueous and thus resides on the RWM for a short time. Therefore, in the present study, we further developed a novel drug delivery system of US-mediated P407-based DEX MBs to achieve efficiently, sustained DEX delivery into the inner ear.

Our previous study first demonstrated the potential clinical application of US-mediated MB cavitation through three courses of USMB treatments via an external auditory canal (transcanal approach) to facilitate drug delivery into the inner ear (Liao et al., 2020). The present study first investigated whether a thermosensitive poloxamer-based MB hydrogel combined with US via a transcanal approach can enhance and extend the sustained release of DEX into the inner ear through one course of USMB treatment without perforations. A steady tissue-mimicking 37 °C heat system was established to estimate the characteristics of MBs in P407 under physiological-like conditions. The cavitation effect induced by the entrance of the ultrasonic wave into the liquid form of the mixture of P407 and MBs (P407-MBs) can increase the permeability of the RWM. Then, the viscosity of the P407-MBs increases as the temperature of the middle ear chamber gradually rises. This change results in a sustained release effect of DEX in P407-MBs across the RWM and reduces the number of repeated injections of P407-MBs via puncture of the tympanic membrane. Previous studies in the literature reported that P407 cannot form a gel regardless of the temperature when the concentration is less than 15% (w/w) (Shau et al., 2015; Zhang et al., 2015; Fakhari et al., 2017). OTO-104, a sustained-release DEX hydrogel that consists of a sterile suspension containing DEX in 16% P407, is prepared for the treatment of otic disorders (Harrop-Jones et al., 2016). Another study demonstrated that no gel was formed at 11.6% w/v and 10.5% w/v P407 concentrations, and the addition of different concentrations of organic cosolvents to P407 solutions affected the viscosity of P407 (Fakhari et al., 2017).

In our present study, the optimal composition of thermosensitive P407 gel for USMB-enhanced inner ear drug delivery was 12.5% P407 in MB and DEX solutions and was used in *in vitro* or *in vivo* permeation drug delivery experiments without cosolvents. Although 10% and 12.5% P407 in MBs and DEX solution were both used in the *in vitro* permeation drug delivery experiments, the viscosity of 10% P407 was not changed following the temperature change and could not form a gel regardless of the temperature (in Figure 5(C) and Table 1). In Franz diffusion experiments, the release rates of biotin-FITC at 6 h and DEX at 3 h in the 12.5% P407-MBs + US groups resulted in 3.00- and 1.52-fold release rates in the 12.5% P407-MB groups. The 12.5% P407-MBs + US group exhibited the highest permeation of DEX, so 12.5% P407-MBs was used in the *in vivo* experiments. In the *in vivo* model of guinea pigs, after the tympanic bulla was filled with DEX in P407-MBs (DEX-P407-MBs), USMB was applied at post-treatment days 1 and 7 induced 109.13% and 66.67% increases in DEX delivery efficiencies, respectively, compared to the group without US. The analysis of perilymph DEX levels and the distribution of DEX uptake in the cochlea confirmed that the delivery of DEX into the inner ear was enhanced and sustained over 7 days by treatment with DEX-P407-MBs and US mediation in the guinea pig model. Moreover, there was no functional or anatomical damage in the middle and inner ear following this treatment. These results demonstrated that US-mediated formulation of DEX-P407-MBs in the middle ear via a transcanal approach can not only enhance DEX delivery through the RWM but also increase drug residence time to maintain the therapeutic level of DEX in the inner ear. Since this design has the characteristics of convenience, noninvasiveness, facilitation of inner ear drug delivery, and sustained drug release, it is promising for application in the treatment of inner ear diseases in future clinical applications.

One previous study reported that P407 gel biodegraded slowly and was almost discharged at 49 days after perfusion in the bullae (Feng et al., 2008). A higher concentration of P407 restricts the elimination of P407 via the Eustachian tube and might cause inflammation and immunologic rejection in the middle ear cavity. In the present study, there was no infiltration of inflammatory cells in the mucosa of the middle ear and no perforation of the tympanic membrane after the middle ear cavity was injected with 12.5% P407-MBs through the tympanic membrane and then exposed to US. Moreover, the application of US-mediated DEX-12.5% P407-MBs could maintain a therapeutic level within the inner ear for at least 7 days. Thus, one course of US-mediated DEX-12.5% P407-MB treatment via a transcanal approach can provide the effect of one week of steroid therapy on the inner ear and be employed as an alternative treatment in patients with inner ear diseases.

## Conclusion

In this study, thermosensitive P407-MBs combined with the US platform were applied to enhance and extend the sustained release of DEX into the inner ear for 7 days through one administration of USMB treatment without adverse effects on the middle and inner ear. The platform achieved an enhanced and sustained release effect of DEX in 12.5% P407-MBs across the RWM without organic solvent and reduced the number of repeated IT injections. The USMB effect can increase RWM permeability, and then, the viscosity of P407-MBs increased as the temperature of the middle ear cavity gradually rose. The lower concentration of P407 in the platform decreased the inflammatory response of the middle ear resulting from the prolonged retention of P407 with a high concentration in the middle ear cavity.

## Author contributions

MWL, CPS, and YCL performed the experiments. AHL and CPS wrote the manuscript with support from CHW. AHL, CPS, HCC, and CHW participated in planning and performing the experiments. AHL, CPS, MWL, and YCL participated in data analysis and interpretation. CPS, AHL, CHW, and HCC made substantial contributions to the conception and design of the research, data collection, and the editing of the manuscript. All of the authors have reviewed the manuscript and approved its final version.
